# Biochanin A Exerts Broad-Spectrum Antiviral Activity Against Coronaviruses via Activating the AMPK/Nrf2/GSH Pathway

**DOI:** 10.3390/microorganisms14040851

**Published:** 2026-04-09

**Authors:** Qisheng Lin, Fan Ji, Haiyan Shen, Jiajing He, Donglan Liu, Fang Li, Ziyu Cheng, Weisan Chen, Fengxiang Zhang, Zifeng Yang, Jianxin Chen

**Affiliations:** 1 Guangdong Provincial Key Laboratory of Veterinary Pharmaceutics Development and Safety Evaluation, College of Veterinary Medicine, South China Agricultural University, Guangzhou 510642, China; lqs@stu.scau.edu.cn (Q.L.); jf20242027010@stu.scau.edu.cn (F.J.); 20232027009@stu.scau.edu.cn (J.H.); czy_0122czy@163.com (Z.C.); 2 Institute of Animal Health, Guangdong Academy of Agricultural Sciences, Guangzhou 510640, China; haiyan_0001@163.com; 3 Guangzhou National Laboratory, Guangzhou International Bio-Island, Guangzhou 510320, China; liu_donglan@gzlab.ac.cn; 4 State Key Laboratory of Respiratory Disease, The First Affiliated Hospital of Guangzhou Medical University, Guangzhou 510182, China; lifang180818@163.com; 5 Department of Biochemistry and Genetics, La Trobe Institute for Molecular Science, La Trobe University, Melbourne, VIC 3086, Australia; weisan.chen@latrobe.edu.au; 6 Key Laboratory for Chemistry and Molecular Engineering of Medicinal Resources (Ministry of Education of China), School of Chemistry and Pharmaceutical Science, Guangxi Normal University, Guilin 541004, China; zhangfengxiangjnu@163.com

**Keywords:** biochanin A, porcine epidemic diarrhea virus, human coronavirus HCoV-OC43, human coronavirus HCoV-229E, antiviral activity, AMPK/Nrf2/GSH pathway

## Abstract

Coronavirus infections pose a significant threat to both human and animal health, causing widespread morbidity, mortality, and substantial economic losses. While vaccines are crucial for prevention, their efficacy is often limited by the high mutation rate of these viruses. This underscores the urgent need for anti-coronavirus drugs, particularly broad-spectrum antiviral agents. In this study, we demonstrated for the first time that Biochanin A (BCA), a bioactive isoflavonoid found in legumes, exhibits broad-spectrum antiviral activity against coronaviruses. BCA potently inhibits porcine epidemic diarrhea virus (PEDV), as well as human coronaviruses HCoV-OC43 and HCoV-229E in *vitro*, with EC_50_ values of 6.90, 2.80 and 15.4 μM, respectively. In a lethal mouse model of HCoV-OC43-induced encephalitis, oral administration of BCA (40–60 mg/kg) significantly improved animal survival and reduced cerebral viral loads. Mechanistic studies revealed that BCA upregulates the AMPK/Nrf2 signaling pathway, thereby increasing expression of the glutamate-cysteine ligase catalytic subunit (GCLC) and enhancing glutathione (GSH) biosynthesis. Our findings identify BCA as a promising host-directed antiviral agent and highlight its therapeutic potential against coronavirus infections.

## 1. Introduction

Coronaviruses (CoVs) are a large family of enveloped, positive-sense single-stranded RNA viruses that infect a wide range of avian and mammalian hosts, including humans. They are classified into four genera: *alpha*-, *beta*-, *gamma*-, and *delta*-coronavirus. While some human coronaviruses cause mild respiratory illnesses, others, such as SARS-CoV-2 [1] and MERS-CoV [2], have demonstrated significant pandemic potential and high pathogenicity. In the veterinary field, CoVs also present a serious threat to livestock health and global food security. A notable example is the porcine epidemic diarrhea virus (PEDV), an *alpha*-coronavirus that causes a devastating enteric disease in swine. PEDV infections may lead to mortality rates approaching 100% in neonatal piglets and substantial economic losses [3].

The continuous emergence and cross-species transmission of coronaviruses underscore the urgent need to develop broad-spectrum antiviral strategies. Current control measures against CoVs such as PEDV rely largely on vaccination. However, high mutation and recombination rates, particularly in the viral spike gene, enable immune evasion and frequently undermine vaccine efficacy. In addition to vaccination, the development of antiviral therapeutics constitutes a crucial complementary strategy for controlling viral diseases. This approach has gained considerable attention during the COVID-19 pandemic, where antiviral agents such as remdesivir [4], molnupiravir [5] and paxlovid [6] have been clinically employed to reduce disease severity. However, the efficacy of existing antiviral drugs against coronaviruses affecting livestock, particularly PEDV, remains largely uninvestigated. Although several natural compounds have shown inhibitory effects against various coronaviruses in *vitro* [7,8,9], the absence of approved specific antivirals for many CoVs, including PEDV, underscores a critical gap in our therapeutic arsenal.

Viral replication and pathogenicity often depend on host cellular proteins and signaling pathways. Modulating these host factors may provide effective strategies to suppress viral infection. Notably, an emerging hallmark of coronavirus infection is the induction of severe oxidative stress. Virus infections, including PEDV and HCoV-OC43, trigger excessive reactive oxygen species (ROS) accumulation, which can be exploited to facilitate viral replication [10,11]. Therefore, targeting host pathways that regulate redox homeostasis represents a compelling antiviral strategy. The cellular AMP-activated protein kinase (AMPK) serves as a central sensor of energy status and redox homeostasis [12]. Upon activation, phosphorylated AMPK induces activation of the transcription factor nuclear factor E2-related factor 2 (Nrf2), the master regulator of the antioxidant response [13], followed by Nrf2 translocation to the nucleus to drive the expression of a battery of cytoprotective genes. Among downstream effectors of Nrf2, glutamate-cysteine ligase catalytic subunit (GCLC), the rate-limiting enzyme in the synthesis of glutathione (GSH), represents the most critical mediator [14]. GSH is the predominant intracellular non-enzymatic antioxidant, essential for neutralizing ROS and maintaining redox balance [15]. As such, the AMPK/Nrf2/GSH pathway represents a promising host-directed pathway, the targeting of which could alleviate virus-induced oxidative stress, thereby inhibiting viral replication.

In the search for novel antiviral compounds, natural flavonoids have attracted increasing attention due to their broad spectrum of biological activities. Among them, genistein, a well-studied isoflavone, has been reported to possess anti-coronavirus activity. To identify effective flavonoids with antiviral activity, this study assessed the anti-coronavirus potential of genistein and its five structurally related analogs. Among the six tested compounds, Biochanin A (BCA), a naturally occurring isoflavonoid in edible legumes, showed superior antiviral efficacy and safety margin, with an EC_50_ value of 6.90 μM against PEDV and a remarkable selectivity index (SI) of 39. Furthermore, we demonstrated that BCA exhibits broad-spectrum anti-coronavirus activities against different subtypes of strains in *vitro*, including *alpha*-subtypes PEDV and HCoV-229E and *beta*-subtype HCoV-OC43. Notably, oral administration of BCA significantly protected mice from lethal encephalitis caused by HCoV-OC43 infection. Mechanistically, we revealed that BCA exerts an anti-coronavirus effect by upregulating the AMPK/Nrf2/GSH pathway in *vitro* and in *vivo*.

BCA is a widely occurring isoflavonoid in edible legumes, particularly chickpeas, a traditional staple food. While antitumor and anti-inflammatory activities of BCA have been established, its anti-coronavirus potential remains reported. Our findings reveal that BCA exerts broad-spectrum anti-coronavirus activity by upregulating the AMPK/Nrf2/GSH signaling pathway. This discovery positions BCA as a promising host-targeted lead compound for combating emerging coronaviruses.

## 2. Materials and Methods

### 2.1. Cells and Viruses

African green monkey kidney cells (Vero cells) and porcine small intestinal epithelial cells (IPEC-DQ) were kindly provided by Professor Jianfeng Zhang from the Institute of Animal Health, Guangdong Academy of Agricultural Sciences. The PEDV-*CV777* strain was propagated in Vero cells in DMEM supplemented with 2% FBS. Human hepatocellular carcinoma cells (Huh-7) and human coronavirus HCoV-OC43 and HCoV-229E strains were generously provided by Professor Jincun Zhao’s research group at the State Key Laboratory of Respiratory Disease, Guangzhou Medical University. HCoV-OC43 or HCoV-229E was propagated in Huh-7 cells in DMEM supplemented with 2% FBS. The virus titer was determined by analyzing the 50% tissue culture infective dose (TCID_50_) using the Reed-Muench assay [16].

### 2.2. Compounds and Reagents

Biochanin A, hinokitiol, dorsomorphin, and buthionine sulfoximine were obtained from MedChemExpress (MCE, Monmouth Junction, NJ, USA). Xanthohumol, reduced glutathione (GSH), and N-acetylcysteine (NAC) were obtained from Pufei De Biotech Co., Ltd. (Chengdu, China). Molnupiravir was sourced from Huateng Pharmaceutical Co., Ltd. (Hunan, China). For in vitro experiments, the above compounds were dissolved in 100% dimethyl sulfoxide (DMSO) and diluted with fresh culture medium prior to the experiments so that DMSO’s final concentration was less than 0.4%. For in vivo experiments, BCA and molnupiravir were dissolved in 0.9% sodium chloride injection using vortex mixing to achieve a homogeneous suspension prior to oral administration.

### 2.3. Cytotoxicity Assay

Cells were seeded into 96-well plates (Corning Inc., New York, NY, USA) and cultured until they formed a confluent monolayer. After washing with sterile PBS (Gibco, Grand Island, NY, USA), serial dilutions of the BCA (MCE, Monmouth Junction, NJ, USA) were added. After 48 h of incubation in a cell culture incubator, the supernatant was removed, and 100 μL of 3-(4,5-dimethylthiozol-2-yl)-3,5-dipheryl tetrazolium bromide (MTT, Sigma-Aldrich, St. Louis, MO, USA) solution (0.5 mg/mL) was added to each well. Cells were incubated in the dark at 37 °C for 4 h. Subsequently, 150 µL of dimethyl sulfoxide (DMSO) was added, and the plate was shaken for 15 min to dissolve the formazan crystals. The optical density (OD) was measured at 490 nm using a microplate reader (Thermo Fisher Scientific, Waltham, MA, USA). The 50% cytotoxic concentration (CC_50_) was determined using GraphPad Prism 8.0 software (GraphPad Software, San Diego, CA, USA) [17].

### 2.4. Indirect Immunofluorescence Assay (IFA)

The sample cells were washed with sterile PBS and fixed with 4% paraformaldehyde (Biosharp, Hefei, China) at room temperature (RT) for 20 min. The cells were then permeabilized with 0.3% Triton X-100 (Solarbio, Beijing, China) at RT for 10 min and blocked with 5% BSA at 37 °C for 1 h. Subsequently, an antiviral antibody (1:1000) was added and incubated at 4 °C overnight. The viral protein antibodies included an anti-PEDV mouse monoclonal antibody (Medgene Labs, Brookings, SD, USA), an anti-HCoV-OC43 rabbit monoclonal antibody and an anti-HCoV-229E rabbit monoclonal antibody (Sino Biological, Beijing, China). After washing, goat anti-mouse IgG H&L (Alexa Fluor^®^ 594) (1:1000) or goat anti-mouse IgG H&L (Alexa Fluor^®^ 488) (1:1000) (Beyotime, Shanghai, China) was added and incubated at 37 °C for 1 h. 4,6-diamidino-2-phenylindole (DAPI, 300 nM) (Sigma-Aldrich, St. Louis, MO, USA) was added and incubated at RT for 10 min for nuclear staining. Fluorescence images were captured using a DMI 4000B fluorescence microscope (Leica, Wetzlar, Germany). Fluorescence intensity of each well was quantified using ImageJ software (ImageJ 1.54r, National Institutes of Health, Bethesda, MD, USA) to determine the 50% effective concentration (EC_50_), and data were analyzed using GraphPad Prism 8.0 [18].

### 2.5. Quantitative Real-Time PCR (qRT-PCR)

Total RNA was extracted according to the instructions of the RNA extraction kit (Fastagen, Shanghai, China). Reverse transcription was performed using a first-strand cDNA synthesis kit (TaKaRa, Dalian, China), followed by quantitative PCR using SYBR Green I qPCR Mix (Genstar, Beijing, China) on the CFX96 Real-time PCR system (Bio-Rad, Hercules, CA, USA). Relative gene expression levels were calculated using the 2^−ΔΔCt^ method [17]. Specific primer sequences are listed in Table 1.

### 2.6. Western Blot

The sample cells were washed with ice-cold PBS and lysed using RIPA lysis buffer containing a protease inhibitor cocktail (Beyotime, Shanghai, China). Lysates were centrifuged at 10,000× *g* for 15 min at 4 °C. The resulting supernatants were collected, mixed with loading buffer, and then incubated at 95 °C for 10 min. Proteins were separated by 12% SDS-PAGE and transferred to PVDF membranes (Millipore, Burlington, MA, USA). Membranes were blocked with 5% skim milk in TBST for 1 h at RT, incubated with primary antibodies overnight at 4 °C, and then with HRP-conjugated goat anti-mouse or anti-rabbit IgG (H-L) secondary antibodies (1:5000) for 1 h at RT. The Odyssey system (LICOR, Lincoln, NE, USA) was used to analyze the protein bands on PVDF membranes [18].

Primary antibodies used in this study were obtained from the following commercial sources: The phospho-AMPKα (Thr172) rabbit monoclonal antibody (D4D6D), HO-1 (E3F4S) rabbit mAb (#43966), and NQO1 (D6H3A) rabbit mAb (#62262) were obtained from Cell Signaling Technology (CST, Danvers, MA, USA). The phospho-GSK3 *beta* (Ser9) antibody (#AF2016), GSK3 *beta* antibody (#AF5016), GAPDH antibody (AF7021) and SOD1 antibody (#AF5198) were obtained from Affinity Biosciences (Jiangsu, China). The Nrf2 (T55136), SQSTM1/p62 antibody (TA5384), xCT antibody (TD12509) and Keap1 antibody (TA5266) were obtained from AbMART (Shanghai, China). The HA tag polyclonal antibody (51064-2-AP), GCLC polyclonal antibody (12601-1-AP), GCLM recombinant monoclonal antibody (82623-1-RR), and GSS polyclonal antibody (15712-1-AP) were obtained from Proteintech Group, Inc. (Wuhan, China). The AMPK *alpha* 1 rabbit monoclonal antibody (AF1627) was obtained from Beyotime (Shanghai, China). The phospho-Nrf2 (Ser40) rabbit monoclonal antibody (#680638) was obtained from Zen BioScience (Chengdu, China).

### 2.7. Endpoint Dilution Assay for Virus Titration

The cell or supernatant samples underwent three freeze–thaw cycles. Supernatants from each well were collected. The samples were serially diluted 10-fold and added to the cells, followed by incubation in a cell culture incubator. After 48 h, cytopathic effects (CPE) were observed under a D-63505 Bio-Optical Inverted Microscope (Leica, Wetzlar, Germany), and TCID_50_ values were then calculated using the Reed and Muench formula [16].

### 2.8. Measurement of Reactive Oxygen Species (ROS) Level

The sample cells were washed with cold PBS and incubated with 5 μM H2DCFDA (MCE, Monmouth Junction, NJ, USA) for 20 min, followed by washing with PBS. For fluorescence microscopy, the stained cells were immediately observed and imaged using a Leica DMI 4000B fluorescence microscope [19]. For quantitative analysis by flow cytometry, the cells were resuspended in PBS after staining and washing. The fluorescence intensity of at least 10,000 cells per sample was subsequently measured using a flow cytometer (CytoFLEX, Beckman Coulter, Brea, CA, USA).

### 2.9. Plasmid Construction and Cell Transfection

Vero cells were transfected with 30 nM of siAMPK, siNrf2 or negative control (siNC) (RiboBio Co., Ltd., Guangzhou, China) using jetPRIME^®^ (Polyplus-transfection, Illkirch, France) according to the manufacturer’s recommendations. At 48 h, the cells were infected with 100 TCID_50_ PEDV and treated with BCA. At 24 hpi, the cells were harvested for Western blot as described above. The siRNA sequences are as follows: siAMPK-1, 5’-caaccatgattgacgatga-3’; siAMPK-2, 5’-gcataccatctcataatag-3’; siAMPK-3, 5’-atggcagaagtatgtagag-3’; siNrf2-1, 5’-ggaguaagucgagaaguauuu-3’; siNrf2-2, 5’-gacgacaugcaacaggauauu-3’; siNrf2-3, 5’-ugacucuggcauuucacuaaa-3’; siNC, 5’-uucuccgaacgugucacgutt-3’. The nuclear factor erythroid 2-related factor 2 (Nrf2) gene (GeneID: XM_038000639.2) was amplified with cDNA from Vero cells and cloned into the pCMV with an HA tag to produce pCMV-Nrf2-HA. Vero cells were transfected with 0.5 μg of pCMV-Nrf2-HA using Lipo8000™ (Beyotime, Shanghai, China) according to the manufacturer’s recommendations. At 48 h, the cells were infected with 100 TCID_50_ PEDV. The cells and relative supernatants were collected at 24 hpi for IFA, qRT-PCR, and the end-point dilution assay as described above [20].

### 2.10. Measurement of Intracellular Glutathione (GSH) Level

Intracellular GSH content was determined using the Reduced Glutathione (GSH) Assay Kit (Nanjing Jiancheng Bioengineering Institute, Nanjing, China), following the manufacturer’s protocol. Briefly, the sample cells were collected and washed with ice-cold PBS, followed by resuspension in PBS and lysing by ultrasonication on ice (200–300 W power, 5 s ON/15 s OFF, repeated 3–5 cycles). An aliquot of the resulting homogenate was immediately mixed with an equal volume of Reagent 1 working solution. The mixture was centrifuged at 4000× *g* for 10 min at 4 °C to remove precipitates. The supernatant was then collected and reacted with the kit’s reagents 2 and 3. After a 5 min incubation at room temperature, the absorbance was measured at 405 nm using a microplate reader. The GSH concentration in the samples was calculated based on the standard curve generated with the kit’s GSH standard and normalized to the total protein concentration of the cell homogenate.

### 2.11. Mouse Experiments

The animal study protocol was approved by the Chinese Laboratory Animal Regulations (Ministry of Science and Technology of the People’s Republic of China) and the National Laboratory Animal Standardization Technical Committee for studies involving animals. The Laboratory Animal Center of South China Agricultural University approved this study, and the approval number is 2025C016.

Specific pathogen-free (SPF) male C57BL/6 (6 weeks old) were purchased from BesTest Bio-Tech Co., Ltd. (Zhuhai, China). To evaluate BCA’s protective effects on survival and body weight in HCoV-OC43-infected mice, animals were intranasally challenged with 1000 TCID_50_ HCoV-OC43 and orally administered BCA (20, 40, or 60 mg/kg) or molnupiravir (20 mg/kg, positive control) at 4 hpi, while the virus control group received an equal volume of 0.9% saline; mice (n = 6) were monitored for 14 days to assess clinical manifestations, survival, and body weight changes. To examine BCA’s effects on viral replication and histopathology, a separate cohort underwent identical infection and treatment protocols before being humanely sacrificed at 7 dpi (n = 6). Brain tissues were collected for qRT-PCR quantification for viral RNA level; Western blot analysis for viral nucleocapsid (N) protein, p-AMPK, AMPK, GCLC and Nrf2 expressions; and hematoxylin and eosin (H&E) staining post 4% paraformaldehyde fixation.

### 2.12. Statistical Analysis

All experiments were performed at least three times. The results were presented as mean standard deviation (SD). Statistical significance was determined by the Student’s *t* test when only two groups were compared or by one-way analysis of variance (ANOVA) when more than two groups were compared. * *p* < 0.05, ** *p* < 0.01, and *** *p* < 0.001 were statistically significant at different levels. ns: no significant difference.

## 3. Results

### 3.1. Biochanin A (BCA) Exhibits Potent Anti-PEDV Activity In Vitro

**Figure 1:**
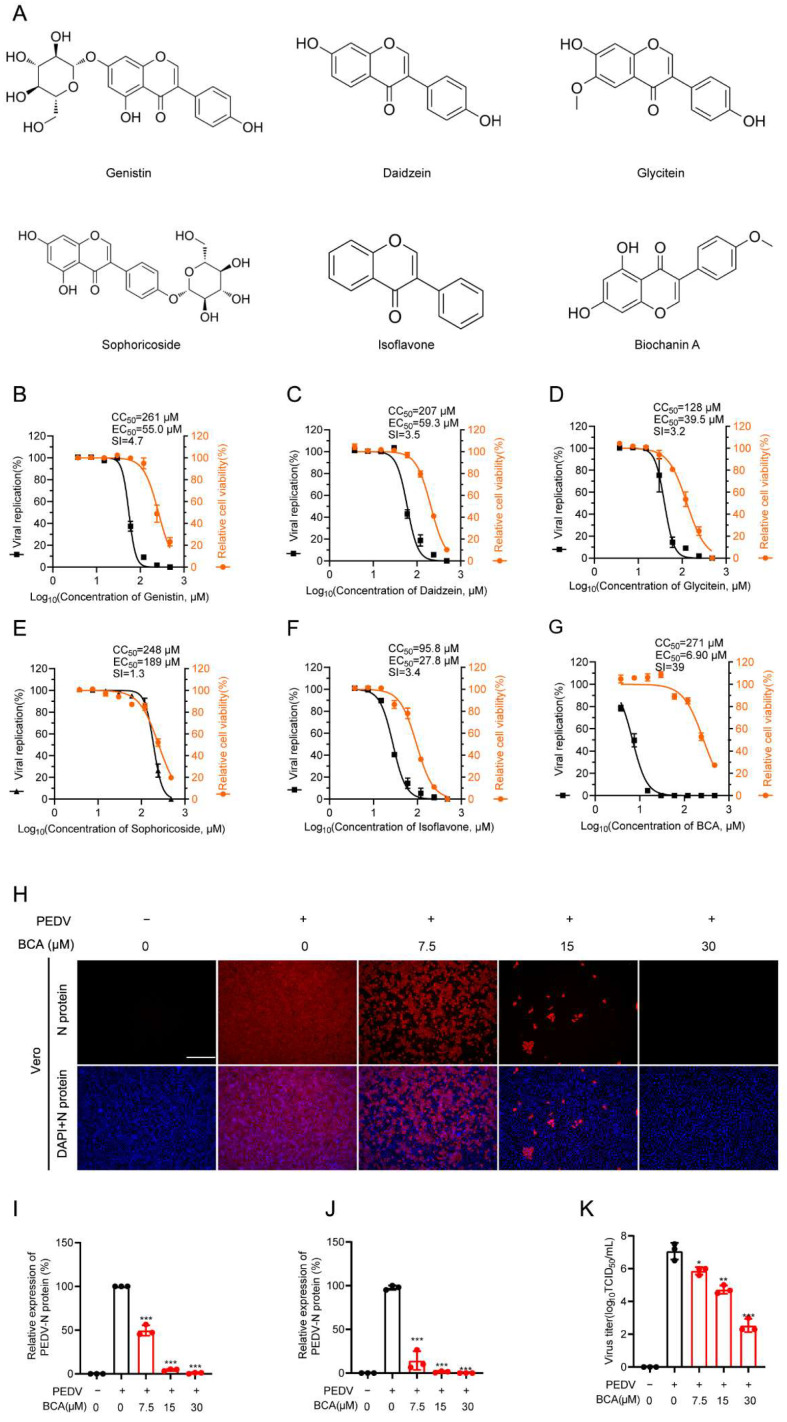
Evaluation of the anti-PEDV activity of six natural flavonoids. (**A**) Chemical structures of genistein and five structurally related analogs. (**B**–**G**) Dose–response curves demonstrated the cytotoxicity (CC_50_) of the six flavonoids and their anti-PEDV effects (EC_50_) in Vero cells. Cytotoxicity of the six flavonoids in Vero cells after 48 h of treatment was assessed by MTT assay to determine the CC_50_. The EC_50_ was evaluated by IFA as described in the Methods. The selective index (SI) was calculated based on CC_50_ and EC_50_ (CC_50_/EC_50_). (**H**) Representative IFA images showed the inhibitory effect of BCA treatment against PEDV replication in Vero cells, reflected by the expression of PEDV nucleocapsid protein. Scale bar: 250 μm. (**I**) Quantification of fluorescence intensity from (**H**). (**J**) Relative viral mRNA levels were determined by qRT-PCR. (**K**) Progeny viral titers were measured by an endpoint dilution assay. Statistical significance is denoted by * *p* < 0.05, ** *p* < 0.01 and *** *p* < 0.001, compared with the DMSO control.

Given the well-documented broad-spectrum antiviral activities of flavonoids, we assessed the anti-PEDV potential of genistein and five structurally related analogs in a search for effective antiviral agents. The chemical structures of these compounds are shown in Figure 1A. We first determined their cytotoxicity in Vero cells and their inhibitory effects on PEDV infection. Using an MTT assay after 48 h of treatment, we determined that the 50% cytotoxic concentration (CC_50_) values for genistein, daidzein, glycitein, sophoricoside, isoflavone, and biochanin A (BCA) were 261, 207, 128, 248, 95.8, and 271 μM, respectively (Figure 1B–G). The CC_10_ values for these six compounds were also calculated and are presented in supplementary Table S1. Subsequently, under non-cytotoxic conditions, the 50% effective concentration (EC_50_) against PEDV infection was established by immunofluorescence assay (IFA), with values of 55, 59.3, 39.5, 189, 27.8, and 6.90 μM for the respective compounds (Figure 1B–G). Among the five evaluated flavonoids, BCA demonstrated the most potent inhibition against PEDV infection and achieved the highest antiviral selective index (SI = CC_50_/EC_50_) of 39. On this basis, it was selected for further investigation.

We next assessed the effect of BCA on PEDV replication by examining viral protein expression, mRNA level, and progeny virus production using IFA, qRT-PCR, and an endpoint dilution assay, respectively. IFA staining of viral nucleocapsid (N) protein demonstrated that BCA dose-dependently inhibited viral protein expression (Figure 1H,I). Consistent with the IFA results, BCA also suppressed viral mRNA expression in a dose-dependent manner (Figure 1J), with an mRNA level decrease of 98.6% at 15 μM. Moreover, BCA significantly reduced the production of progeny virus, with a viral titer decrease of 2.4 log TCID_50_ at 15 μM (Figure 1K). Taken together, these results demonstrate that BCA exerts a potent and dose-dependent inhibitory effect on PEDV replication in vitro.

### 3.2. BCA Demonstrates Broad-Spectrum Anti-Coronavirus Activity

**Figure 2:**
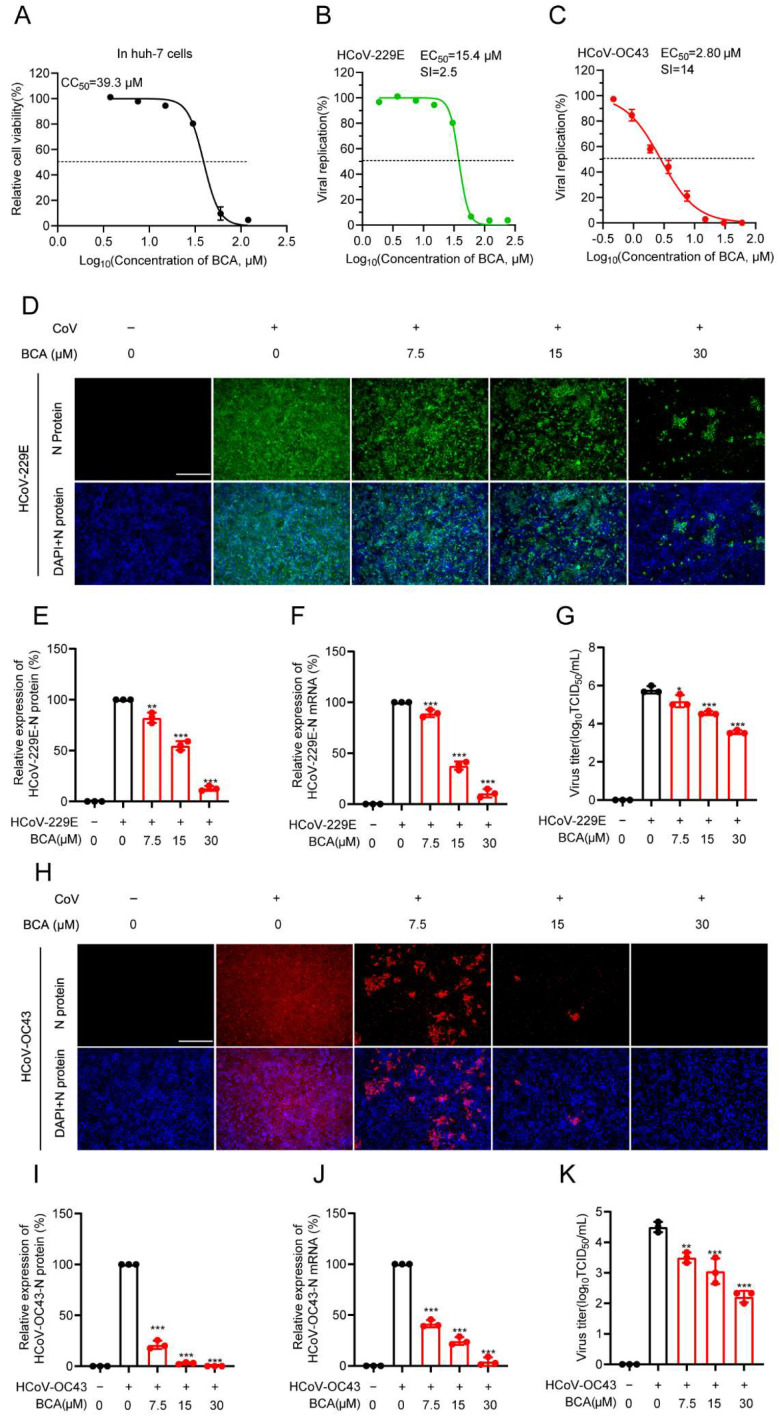
BCA exhibits significant antiviral activity against the *alpha*-coronavirus HCoV-229E and the *beta*-coronavirus HCoV-OC43. (**A**) Cytotoxicity of BCA in Huh-7 cells after 72 h of treatment was assessed by MTT assay to determine the CC_50_. (**B**,**C**) The EC_50_ value was evaluated by IFA following HCoV-229E or -OC43 infection for 2 h and BCA treatment for 72 h. SI = CC_50_/EC_50_. (**D**–**H**) Representative IFA images showed the inhibitory effect of BCA treatment on viral replication reflected by the N protein expression of HCoV-229E or -OC43 in Huh-7 cells. Scale bar: 250 μm. (**E**–**I**) Quantification of fluorescence intensity from (**D**) and (**H**), respectively. (**F**–**J**) Relative viral mRNA levels of HCoV-229E or -OC43 were determined by qRT-PCR. (**G**–**K**) Progeny viral titers of HCoV-229E or -OC43 were measured by an endpoint dilution assay. Statistical significance is denoted by * *p* < 0.05, ** *p* < 0.01 and *** *p* < 0.001, compared with the DMSO control.

To determine whether BCA possesses broad-spectrum anti-coronavirus activity, we evaluated its efficacy against the *alpha*-coronavirus HCoV-229E and the *beta*-coronavirus HCoV-OC43. As shown in Figure 2A, BCA exhibited a mild cytotoxicity with a CC_50_ value of 38.3 μM in Huh-7 cells after 72 h of treatment. The EC_50_ values against HCoV-229E and HCoV-OC43, as measured by IFA in Huh-7 cells, were 15.4 μM and 2.8 μM, respectively (Figure 2B,C), yielding selective index (SI) values of 2.5 and 13.7. We further examined the effect of BCA on viral replication. IFA results revealed that BCA reduced N protein expression in both HCoV-229E and HCoV-OC43 in a dose-dependent manner (Figure 2D–I). Results of qRT-PCR and viral titer analysis confirmed that BCA significantly suppressed viral mRNA levels (Figure 2F,J) and progeny virus production (Figure 2G,K). Taken together, these results indicate that BCA possesses broad-spectrum anti-coronaviral activity, effectively inhibiting both *alpha*- and *beta*-coronaviruses.

### 3.3. Network Pharmacology Analysis Suggests That BCA May Suppress Coronavirus Infection by Regulating the Nrf2-Mediated Antioxidant Pathway

**Figure 3:**
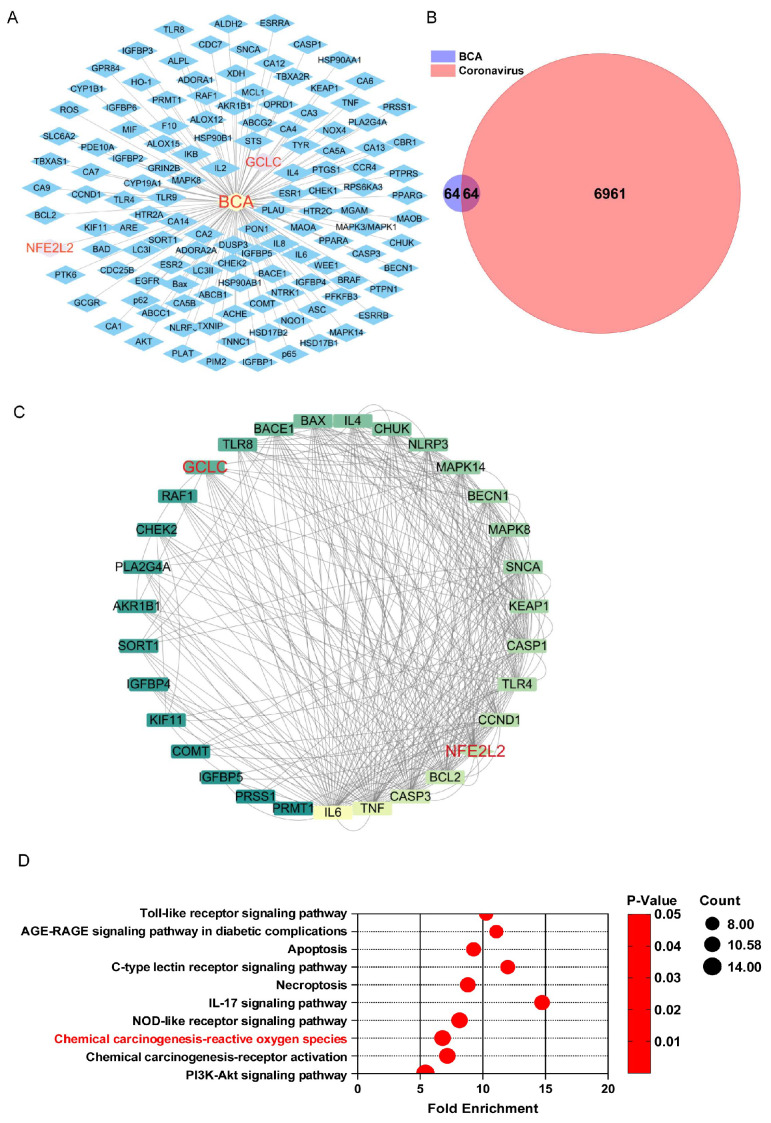
The network pharmacology analysis suggested that BCA may suppress coronavirus infection by regulating the Nrf2-mediated antioxidant pathway. (**A**) Compound-target network of BCA; (**B**) Venn diagram of BCA targets and coronavirus targets; (**C**) protein–protein interactions of 64 overlapped targets between BCA and coronavirus; (**D**) top 10 KEGG signal pathways of 64 overlapped targets between BCA and coronavirus.

A total of 128 targets of BCA were retrieved from the Swiss Target Prediction database, including Nrf2 (also named NEF2L2) and GCLC (Figure 3A). Among them, 64 targets were shown in 7025 coronavirus infection-related targets collected in Gene Cards (Figure 3B), indicating that these were the potential affected targets for BAC to suppress coronavirus infections. Further, the protein–protein interactions of 64 overlapped targets between BCA and coronavirus infection were generated from the STRING database. Thirty-two targets formed the main network interactions, including IL-6, TNF, CASP3, BCL2, NEF2L2, etc., with the interaction degree value more than 4, and were regarded as the potential core targets for BCA to suppress coronavirus infections (Figure 3C). Moreover, the KEGG analysis in the DAVID database indicated that the PI3K-Akt signaling pathway, chemical carcinogenesis-receptor activation, and chemical carcinogenesis-reactive oxygen species were the top 3 signal pathways affected by BCA to achieve its anti-coronavirus effects. According to the predicted targets and previous work, we speculated that modulating the reactive oxygen species might be one of the core pathways for BCA to suppress coronavirus infections.

### 3.4. BCA Alleviates PEDV-Induced Oxidative Stress

**Figure 4:**
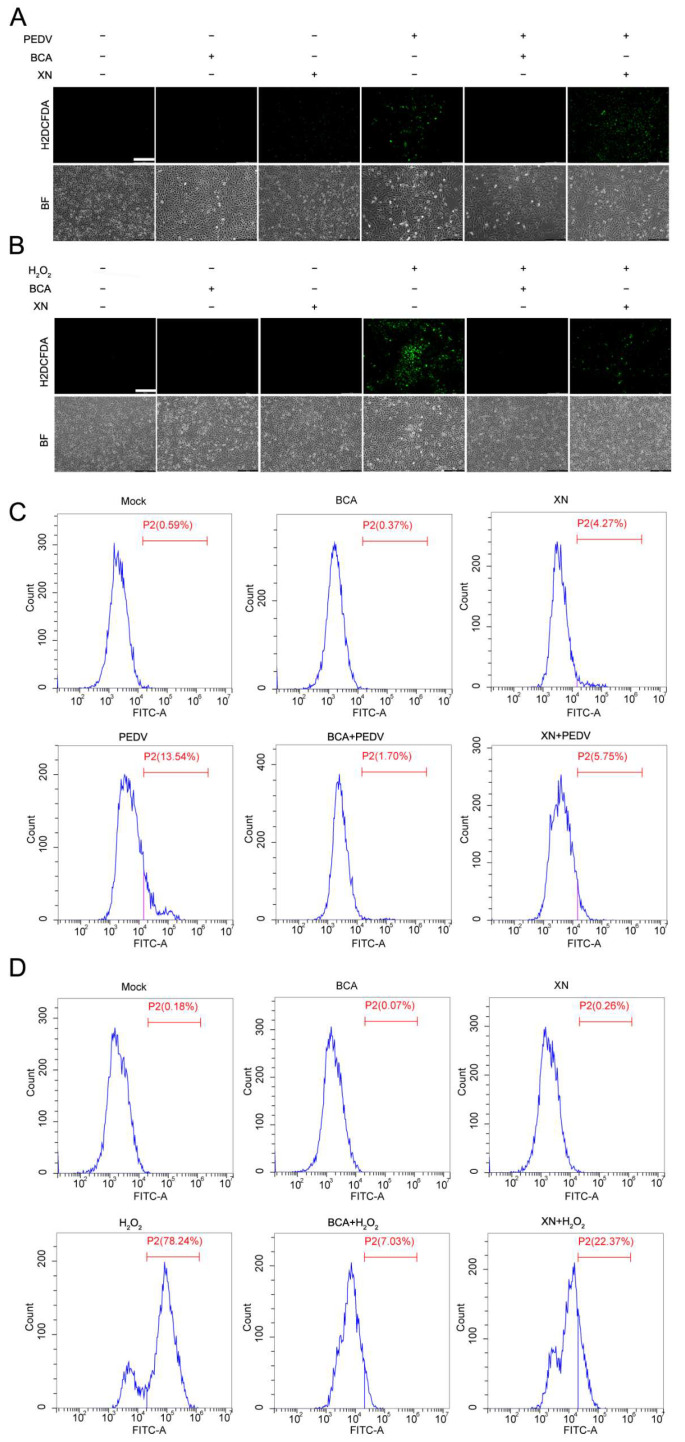
BCA alleviates PEDV- and H_2_O_2_-induced oxidative stress. (**A**,**B**) Microscopy analysis of ROS-scavenging effects of BCA against PEDV- and H_2_O_2_-induced oxidative stress. Vero cells were infected with 100 TCID_50_ of PEDV for 2 h and followed by treatment with DMSO or 15 μM BCA for 24 h. (**A**) Vero cells were treated with 1 mM H_2_O_2_ and DMSO or 15 μM BCA for 2 h. (**B**) The cells were incubated with the 5 μM H2DCFDA fluorescent probe for 20 min, washed, and imaged using a fluorescence inverted microscope. Scale bar: 250 μm. (**C**,**D**) Flow cytometric quantification of intracellular ROS levels. (**C**) Vero cells were infected with 100 TCID_50_ of PEDV for 2 h, followed by treatment with DMSO or 15 μM BCA for 24 h. (**D**) Cells were treated with 1 mM H2O2 together with DMSO or 15 μM BCA for 2 h. After treatment, cells were stained with 5 μM H2DCFDA for 20 min and analyzed by flow cytometry. Data are presented as the percentage of ROS-positive cells relative to the respective control.

Previous studies have shown that PEDV infection induces intracellular ROS production to facilitate its replication [11]. Our network pharmacology analysis shown in Figure 3 suggested that the antiviral activity of BCA might be related to its antioxidant activity. Therefore, we investigated the effect of BCA on PEDV-induced oxidative stress by measuring intracellular ROS levels in PEDV-infected cells. In addition, H_2_O_2_ was used as a positive control to validate the ROS-scavenging specificity of BCA. Intracellular ROS levels were detected using the fluorescent probe H2DCFDA, which emits green fluorescence proportional to the amount of ROS. The results revealed that BCA treatment significantly suppressed the accumulation of intracellular ROS elicited by both PEDV infection (Figure 4A) and exogenous H_2_O_2_ stimulation (Figure 4B), validating its ROS-scavenging activity under pathologically relevant conditions. Moreover, flow cytometry results also confirmed that BCA could inhibit the increased ROS levels induced by PEDV infection (Figure 4C) or exogenous H_2_O_2_ stimulation (Figure 4D). Xanthohumol (XN), a compound previously reported to exhibit significant antiviral activity against PEDV through enhancing antioxidant pathways [21], was used as the positive control.

### 3.5. Nrf2 Activation Is Essential for the Anti-PEDV Activity of BCA

**Figure 5:**
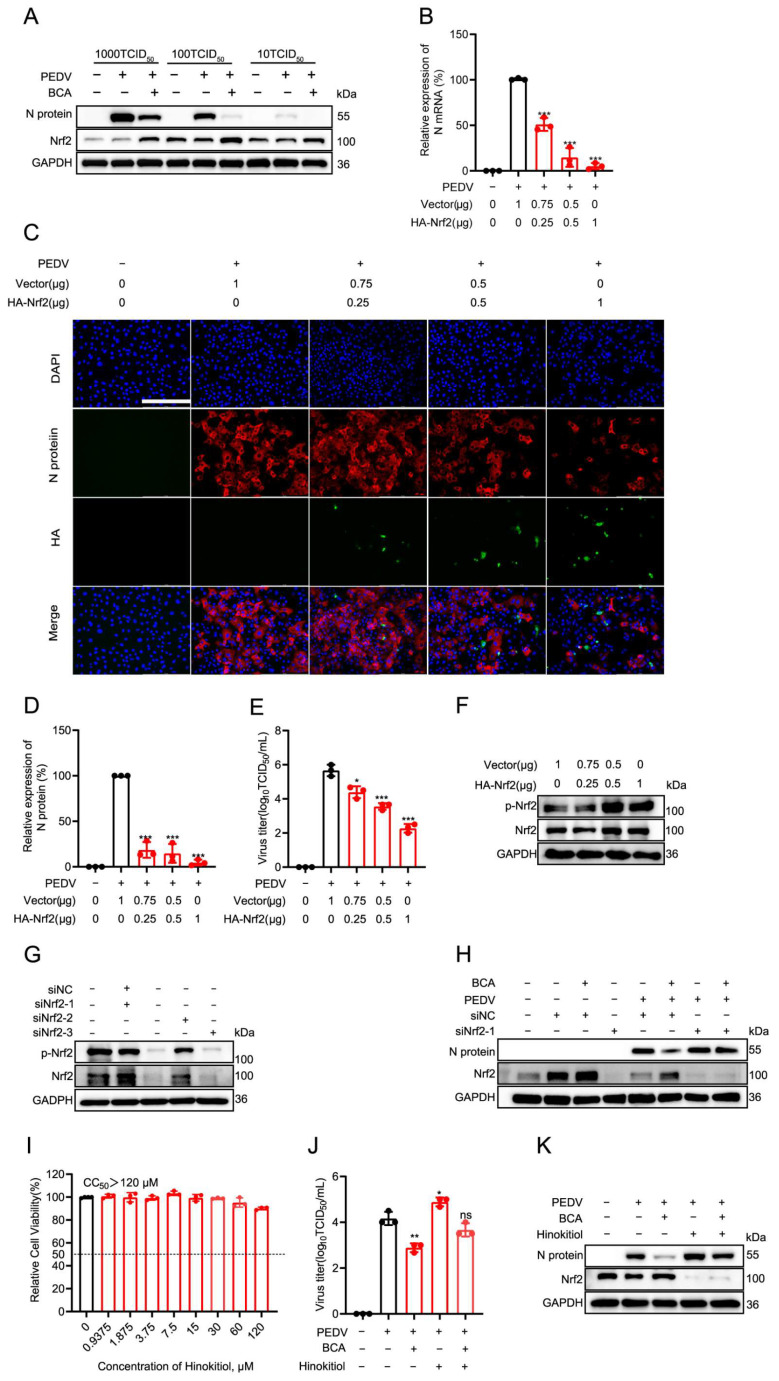
Upregulation of Nrf2 suppresses PEDV replication. (**A**) Vero cells were infected with PEDV for 2 h at 10, 100, or 1000 TCID_50_, followed by treatment with 15 μM BCA or DMSO for 24 h, and then the protein expression levels of N protein and Nrf2 in the cells were analyzed using Western blot. (**B**–**E**) The effect of Nrf2 overexpression on PEDV replication. Vero cells were transfected with HA-Nrf2 (0.25, 0.5, or 1 μg) or an empty vector for 48 h, followed by infection with 100 TCID_50_ PEDV for 2 h. Viral replication was evaluated at 24 hpi. (**B**) Relative PEDV mRNA levels were determined by qRT-PCR. (**C**) Representative IFA images showed the effects of HA-Nrf2 treatment on the expression of the PEDV N protein in Vero cells. Scale bar: 250 μm. (**D**) Quantification of fluorescence intensity from (**C**). (**E**) PEDV progeny viral titers were measured by an endpoint dilution assay. (**F**) Effect of Nrf2 manipulation on p-Nrf2 levels. Vero cells were transfected with increasing amounts of HA-Nrf2 plasmid, and protein levels of Nrf2 and p-Nrf2 were analyzed by Western blot. (**G**) Screening of siRNA fragments targeting Nrf2. Three individual Nrf2 siRNAs (siNrf2-1, siNrf2-2, siNrf2-3) were transfected into Vero cells. After 72 h, protein levels of Nrf2 were measured by Western blot. (**H**) Knockdown of Nrf2 reverses the antiviral effect of BCA. Vero cells were transfected with siNrf2-1 for 48 h, infected with 100 TCID_50_ of PEDV for 2 h, and then treated with 15 μM BCA for 24 h. Protein expression levels of N protein and Nrf2 were measured by Western blot. (**I**) Cytotoxicity of Hinokitiol (a specific Nrf2 expression inhibitor) in Vero cells after 48 h of treatment was assessed by MTT assay to determine the CC_50_. (**J**,**K**) Hinokitiol reversed the antiviral effect of BCA. Vero cells were infected with 100 TCID_50_ of PEDV for 2 h, followed by treatment with Hinokitiol (15 μM) or BCA (15 μM) or their combination for 24 h. The antiviral effects were evaluated by determining progeny viral titers (**J**) and viral N protein levels (**K**). Statistical significance is denoted by * *p* < 0.05, ** *p* < 0.01 and *** *p* < 0.001; ns: no significant difference, compared with the DMSO control.

We then investigated whether BCA exerts its antioxidant effect through the transcription factor Nrf2, a central regulator of cellular antioxidant responses. Western blot analysis showed that treatment with 15 μM BCA for 48 h significantly upregulated Nrf2 expression across a range of PEDV infectious doses (10, 100, and 1000 TCID_50_; Figure 5A), suggesting that BCA may exert its antioxidant activity by enhancing Nrf2 protein levels. To further clarify the role of Nrf2 in the antiviral process, we next examined the effect of Nrf2 overexpression on viral replication. Vero cells were transfected with increasing amounts of an HA-Nrf2 plasmid, followed by PEDV infection. qRT-PCR results showed that HA-Nrf2 reduced the expression of the PEDV N mRNA in a dose-dependent manner (Figure 5B). Consistent with this, Nrf2 overexpression also led to a significant, dose-dependent suppression of viral N protein levels (Figure 5C,D) and progeny virus production (Figure 5E). To establish the functional requirement of Nrf2 for BCA’s antiviral activity, we knocked down Nrf2 expression using siRNA. To evaluate the effect of Nrf2 manipulation on its phosphorylation status, we examined p-Nrf2 levels in cells transfected with HA-Nrf2 or siNrf2. Overexpression of HA-Nrf2 increased both total Nrf2 and p-Nrf2 levels in a dose-dependent manner (Figure 5F). Following a screen of three siRNA sequences, siNrf2-1 was selected for its potent suppression of Nrf2 protein expression (Figure 5G). Transfection with siNrf2-1 effectively reversed the BCA-induced upregulation of Nrf2 and, concomitantly, restored PEDV N protein expression (Figure 5H), indicating that the antiviral effect of BCA is dependent on Nrf2. Furthermore, we utilized hinokitiol, a specific pharmacological inhibitor of Nrf2, to confirm the effect of Nrf2 on PEDV infection. Treatment with hinokitiol at a non-cytotoxic concentration (CC_50_ >120 μM; Figure 5I) significantly increased PEDV progeny virus titer and reversed the antiviral effect of BCA (Figure 5J). Western blot analysis confirmed that hinokitiol reduced Nrf2 expression, promoted PEDV N protein production, and abolished the BCA’s ability to induce Nrf2 and inhibit PEDV replication (Figure 5K). Collectively, these results demonstrate that Nrf2 acts as a host antiviral factor and that the anti-PEDV activity of BCA is mechanistically linked to its ability to enhance Nrf2 expression.

**Figure 6:**
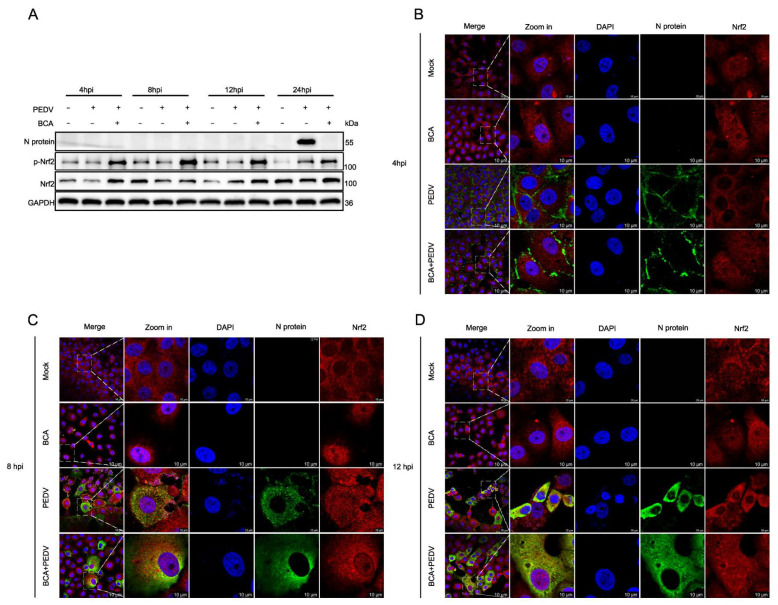
BCA increases Nrf2 expression and its nuclear translocation. (**A**) Vero cells were treated with 15 μM BCA or DMSO for different periods of time (4, 8, 12, or 24 h) following PEDV infection for 2 h at 100 TCID_50_, and then the protein expression levels of N protein, p-Nrf2 and Nrf2 were analyzed. (**B**–**D**) Confocal analysis of BCA-induced Nrf2 nuclear translocation. Vero cells, with or without 10000 TCID_50_ PEDV infection, were treated with 15 μM BCA for 4, 8, or 12 h. Nrf2 localization was observed by confocal microscopy. Scale bar: 10 μm.

A time-course experiment further revealed that BCA markedly enhanced p-Nrf2 and Nrf2 levels as early as 4 hpi, with sustained upregulation observed at 8, 12, and 24 hpi (Figure 6A). Importantly, confocal microscopy analysis revealed a time-dependent alteration in the subcellular localization of Nrf2 upon PEDV infection (Figure 6B–D). At 4 hpi, Nrf2 was predominantly retained in the cytoplasm in PEDV-infected cells (Figure 6B). Clear nuclear translocation of Nrf2 was observed at 8 hpi (Figure 6C), whereas by 12 hpi, the protein had largely re-localized to the cytoplasm (Figure 6D). This dynamic redistribution suggests that PEDV may initially delay Nrf2 nuclear import during early infection and subsequently suppress its nuclear accumulation or promote its nuclear export at later stages of replication. Strikingly, BCA treatment consistently enhanced Nrf2 nuclear translocation at all time points examined (4, 8, and 12 hpi) in both mock- and PEDV-infected cells. These observations provide direct visual evidence that BCA sustains activation of the Nrf2 signaling pathway throughout PEDV infection. Collectively, these data indicate that BCA mitigates PEDV-induced oxidative stress by enhancing Nrf2 expression and increasing its nuclear translocation.

### 3.6. BCA Upregulates the AMPK/Nrf2 Signaling Pathway to Suppress PEDV Replication

**Figure 7:**
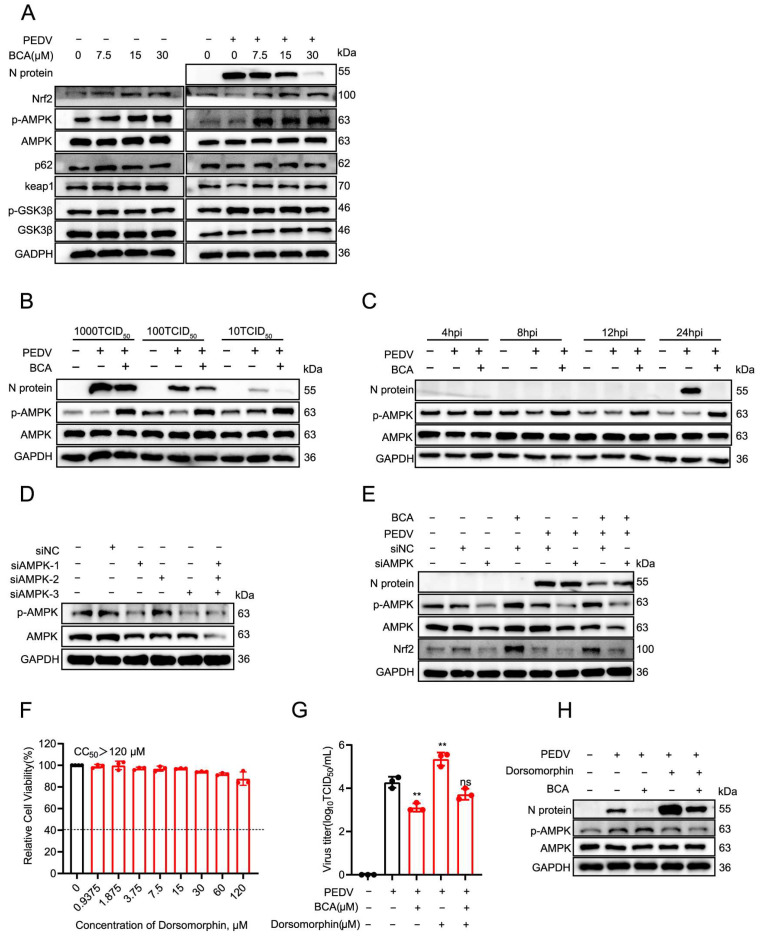
BCA upregulates the AMPK/Nrf2 pathway to inhibit PEDV replication. (**A**) Effects of BCA on upstream regulators of Nrf2. Vero cells were infected with 100 TCID_50_ PEDV for 2 h and then treated with 15 μM BCA for 24 h. Protein expression levels of N protein, p-AMPK, AMPK, p62, keap1, GSK3β, p-GSK3β and Nrf2 were measured by Western blot. (**B**) Vero cells were infected with PEDV at different doses (10, 100, or 1000 TCID_50_) for 2 h, followed by treatment with 15 μM BCA or DMSO. At 24 hpi, protein expression levels of N protein, p-AMPK, and AMPK were analyzed using Western blot. (**C**) Vero cells were infected with 100 TCID_50_ PEDV for 2 h and then treated with 15 μM BCA or DMSO for different periods of time (4, 8, 12, or 24 h). Protein expression levels of N protein, p-AMPK and AMPK were analyzed. (**D**) Screening of siRNA fragments targeting AMPK. Three individual AMPK siRNAs (siAMPK-1, siAMPK-2, and siAMPK-3) and their mixture (siAMPK (1 + 2 + 3)) were transfected into Vero cells. After 72 h, protein levels of AMPK and p-AMPK were detected by Western blot. (**E**) Knockdown of AMPK reversed the antiviral effect of BCA. Vero cells were transfected with siAMPK (1 + 2 + 3) for 48 h, infected with 100 TCID_50_ of PEDV for 2 h, and then treated with 15 μM BCA or DMSO for 24 h. Protein expression levels of N protein, p-AMPK, AMPK and Nrf2 were measured by Western blot. (**F**) Cytotoxicity of Dorsomorphin (a selective, ATP-competitive AMPK inhibitor) in Vero cells after 24 h of treatment was assessed by MTT assay to determine the CC_50_. (**G**,**H**) Dorsomorphin reversed the antiviral effect of BCA. Vero cells were infected with 100 TCID_50_ of PEDV for 2 h, followed by treatment with Dorsomorphin (15 μM) or BCA (15 μM) or their combination for 24 h. The antiviral effects were evaluated by determining progeny viral titers (**G**) and viral N protein levels (**H**). Statistical significance is denoted by ** *p* < 0.01; ns: no significant difference, compared with the DMSO control.

We subsequently examined the effect of BCA on known upstream regulators of Nrf2, including AMPK, p62, and GSK3β. Interestingly, BCA treatment significantly increased the phosphorylation of AMPK (p-AMPK) but had no notable effect on p62 or GSK3β levels (Figure 7A), indicating a potential role of the AMPK pathway in BCA-induced Nrf2 upregulation. Western blot analysis further confirmed that treatment with 15 μM BCA significantly upregulated p-AMPK expression across a range of PEDV infectious doses (10, 100, and 1000 TCID_50_; Figure 7B). A time-course experiment revealed that BCA markedly enhanced p-AMPK levels as early as 4 hpi, with sustained upregulation observed at 8, 12, and 24 hpi (Figure 7C), suggesting that p-AMPK activation is an early cellular response to BCA treatment.

To further investigate whether BCA directly binds to AMPK, we performed CETSA and molecular docking analysis. CETSA results showed that BCA treatment did not alter the thermal stability of AMPK (Supplementary Figure S1A); molecular docking revealed a low binding affinity (−6.5 kcal/mol) between BCA and AMPK, with no stable hydrogen bond interactions formed at the AMPK active site (Supplementary Figure S1B). These results suggest that BCA does not directly bind to AMPK to activate its phosphorylation. To further elucidate the functional relationship between AMPK and Nrf2, we knocked down AMPK expression using siRNA. Through a screen of three individual siRNAs, we identified a pooled combination (siAMPK (1 + 2 + 3)) that effectively reduced the levels of both AMPK and p-AMPK (Figure 7D). Knockdown of AMPK significantly reduced Nrf2 expression, indicating that AMPK acts upstream of Nrf2. Furthermore, AMPK knockdown reversed the BCA-induced upregulation of both p-AMPK and Nrf2 and restored viral replication, reflected by increased PEDV N protein expression (Figure 7E). To pharmacologically validate these findings, we used dorsomorphin, a selective AMPK inhibitor [22]. Treatment with a non-cytotoxic concentration of dorsomorphin (CC_50_ > 120 μM; Figure 7F) significantly increased PEDV progeny virus titers and reversed the antiviral effect of BCA (Figure 7G). Western blot analysis confirmed that dorsomorphin reduced p-AMPK expression, promoted PEDV replication, and counteracted the ability of BCA’s boost on the expression of p-AMPK and inhibition against the viral replication (Figure 7H). Collectively, these results demonstrate that BCA upregulates the AMPK/Nrf2 signaling pathway to exert its anti-PEDV activity.

### 3.7. BCA Inhibits PEDV Replication Through Nrf2-Mediated Glutathione Biosynthesis

**Figure 8:**
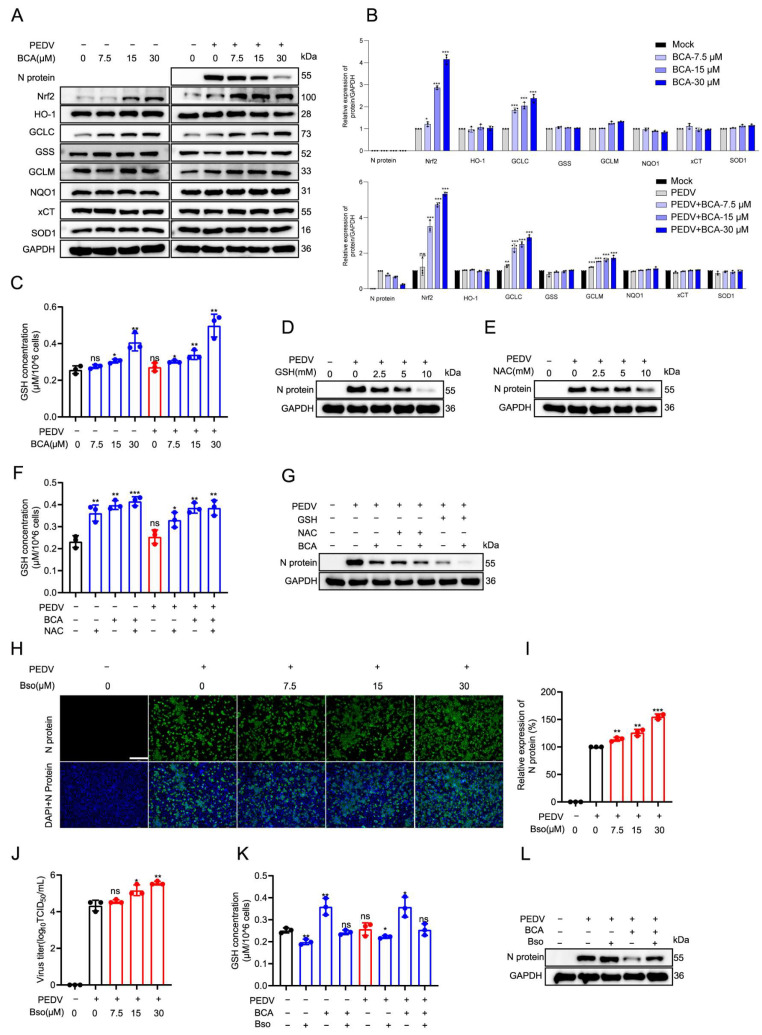
BCA exerts its antiviral effect via Nrf2-mediated GSH enhancement. (**A**) Effects of BCA on the expression of Nrf2 downstream factors. Vero cells, with or without 100 TCID_50_ PEDV infection, were treated with BCA (7.5, 15 and 30 μM) for 24 h. Expression levels of proteins, including viral N protein, Nrf2 and its downstream factors, were measured by Western blot. (**B**) Quantitative results of (**A**). (**C**) Effects of BCA and PEDV infection on intracellular GSH levels. Vero cells, with or without 100 TCID_50_ PEDV infection, were treated with BCA (7.5, 15 and 30 μM) for 24 h. The cells were harvested and lysed by ultrasonication in PBS on ice, and the supernatant was collected by centrifugation. GSH content was measured spectrophotometrically after reaction using a chromogenic kit, including DTNB (5,5’-Dithiobis (2-nitrophenylacetic acid)). (**D**,**E**) Antiviral effects of exogenous addition of GSH and its biosynthetic precursor NAC. Vero cells were infected with 100 TCID_50_ PEDV for 2 h and then treated with exogenous GSH (2.5, 5 and 10 mM) (**D**) or NAC (2.5, 5 and 10 mM) (**E**) for 24 h. Viral N protein expression level was measured by Western blot. (**F**) Effects of NAC and BCA on intracellular GSH levels. Vero cells were infected with 100 TCID_50_ PEDV for 2 h, followed by treatment with 5 mM NAC or 15 μM BCA or their combination for 24 h. Intracellular GSH content was then measured using the chromogenic kit including DTNB. (**G**) Antiviral effects of exogenous addition of GSH, NAC and BCA. Vero cells were infected with 100 TCID_50_ PEDV for 2 h, followed by treatment with 5 mM NAC, or 5 mM GSH, or their combination with 15 μM BCA for 24 h. Viral N protein expression level was measured by Western blot. (**H**–**J**) Promotion of GSH inhibitor buthionine sulfoximine (Bso) on PEDV replication. Vero cells were infected with 100 TCID_50_ PEDV for 2 h and then treated with Bso (7.5, 15 and 30 μM) for 24 h. (**H**) Viral N protein expression level was measured by IFA. Scale bar: 250 μm. (**I**) Quantification of fluorescence intensity from (**H**). (**J**) Progeny viral titer was measured by an endpoint dilution assay. (**K**,**L**) Bso reversed the BCA-induced increase in GSH level and the antiviral effect. Vero cells were infected with 100 TCID_50_ PEDV for 2 h, followed by treatment with 15 μM Bso or 15 μM BCA or their combination for 24 h. Intracellular GSH content was then measured (**K**), and viral N protein expression level was measured by Western blot (**L**). Statistical significance is denoted by * *p* < 0.05, ** *p* < 0.01 and *** *p* < 0.001; ns: no significant difference, compared with the DMSO control.

To identify the specific downstream effectors through which Nrf2 activation mediates the antiviral activity of BCA, we examined the expression of several well-characterized Nrf2-regulated proteins. Western blot analysis demonstrated that BCA specifically and dose-dependently upregulated the expression of GCLC (glutamate-cysteine ligase catalytic subunit), the rate-limiting enzyme in glutathione (GSH) biosynthesis. In contrast, the expression levels of the other Nrf2 downstream effectors, including HO-1, GSS, GCLM, NQO1, xCT, and SOD1, remained unaffected (Figure 8A,B). These findings suggested that GSH might serve as a key downstream mediator of BCA’s antiviral effect. Consistent with the upregulation of GCLC, BCA treatment significantly increased intracellular GSH levels in a dose-dependent manner in both mock- and PEDV-infected cells (Figure 8C). To identify the antiviral potential of GSH, we supplemented the culture medium with exogenous GSH or N-acetylcysteine (NAC), a precursor for GSH synthesis. Both treatments significantly suppressed PEDV N protein expression in a dose-dependent manner (Figure 8D,E), confirming that elevated GSH levels indeed inhibit viral replication. NAC treatment alone effectively increased intracellular GSH content (Figure 8F), demonstrating its role in boosting the cellular antioxidant pool. Furthermore, co-treatment with NAC and BCA exerted a stronger inhibitory effect on PEDV replication than either compound used alone (Figure 8G), suggesting complementary mechanisms in activating the cellular antioxidant defense.

To further substantiate the essential role of GSH in restricting PEDV replication, we employed buthionine sulfoximine (Bso), a specific inhibitor of GSH synthesis. Bso treatment dose-dependently promoted PEDV replication, as evidenced by increased viral N protein expression (Figure 8H,I) and elevated progeny virus titers (Figure 8J). Importantly, Bso effectively abolished the BCA-induced increase in intracellular GSH (Figure 8K) and significantly reversed the antiviral activity of BCA (Figure 8L). Collectively, these results demonstrate that BCA exerts its anti-PEDV activity primarily through the AMPK/Nrf2/GSH signaling pathway, wherein Nrf2-dependent upregulation of GCLC enhances GSH biosynthesis, creating an intracellular environment that restricts viral replication.

### 3.8. Oral Administration of BCA Upregulates the AMPK/Nrf2 Signaling Pathway In Vivo Provides Significant Protection Against HCoV-OC43 Infection in Mice

**Figure 9:**
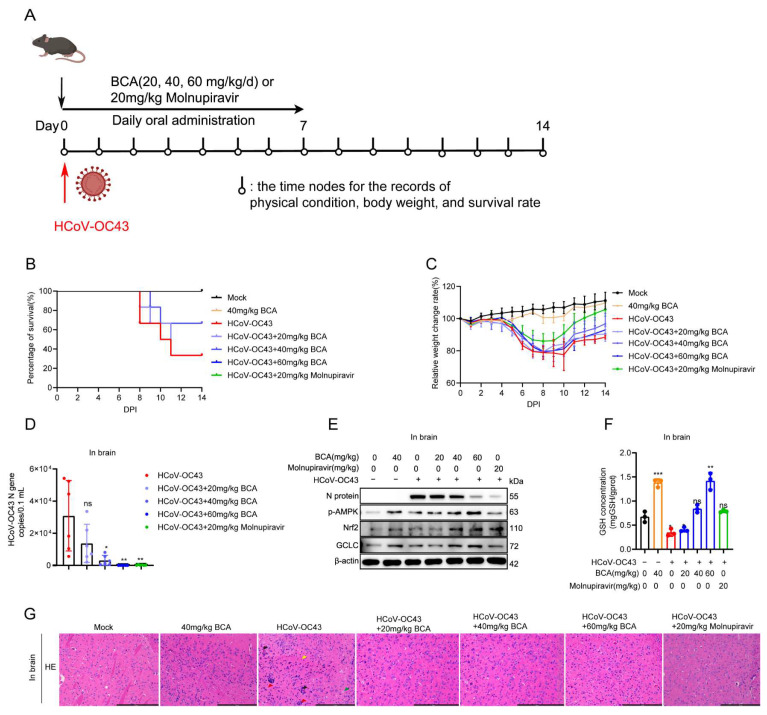
BCA alleviates lethal HCoV-OC43–induced encephalitis via upregulating the AMPK/Nrf2/GSH pathway in mice. (**A**) Experimental timeline: C57BL/6 mice were intranasally inoculated with 1000 TCID_50_ HCoV-OC43 at 0 dpi. Treatment with BCA (20, 40, or 60 mg/kg) or molnupiravir (20 mg/kg), administered orally once daily for 7 consecutive days, was initiated at 4 hpi. Brains were collected at 7 dpi. (**B**) Animal survival curves. (**C**) Animal body weight change. For each brain, one hemisphere was divided for Western blot (homogenized in RIPA buffer) and viral load quantification (homogenized in RA2 lysis buffer for RNA extraction), while the contralateral hemisphere was used for GSH measurement according to the manufacturer’s protocol. (**D**) Cerebral viral loads (HCoV-OC43 N gene copies/0.1 mL). (**E**) Western blot analysis for the expression level of p-AMPK, Nrf2, and GCLC in brain tissues at 7 dpi in each group; β-actin was used as the loading control. (**F**) GSH content in mouse brain tissues. (**G**) H&E-stained hippocampal sections in mouse brain tissues: nuclear shrinkage and degeneration (green arrows), aggregation (red arrows), congestion (black arrows), and vacuolation (yellow arrows). Scale bars: 250 μm. Statistical significance is denoted by * *p* < 0.05, ** *p* < 0.01 and *** *p* < 0.001; ns: no significant difference, compared with the DMSO control.

Having established BCA’s antiviral mechanism in vitro, we next assessed its efficacy in vivo using an HCoV-OC43-infected mouse model. Previous studies reported that HCoV-OC43 induces fatal encephalitis in mice. Molnupiravir, a clinically approved broad anti-coronaviral drug, was used as a positive control [5]. As outlined in Figure 9A, C57BL/6 mice were intranasally inoculated with 1000 TCID_50_ of HCoV-OC43 and treated orally with BCA (20, 40, or 60 mg/kg) or molnupiravir (20 mg/kg) once daily for 7 days, starting at 4 hpi. All animals were monitored for 14 days with daily assessment of clinical status, body weight, and survival. For virological and pathological analyses, all mice were humanely sacrificed at 7 dpi, with brain tissues collected. Following infection, mice developed characteristic clinical signs of encephalitis, including ataxia, lethargy, hunched posture, and reduced appetite. Significant weight loss in HCoV-OC43-infected mice initiated at 5 dpi. In the virus infection group, mice began to die at 8 dpi. Notably, a daily oral dose of 40 or 60 mg/kg of BCA effectively protected mice against lethal HCoV-OC43 infection (Figure 9B). While the virus infection group exhibited only 33.3% survival by 14 dpi, oral administration of BCA at 40 or 60 mg/kg increased survival rates to 66.7%. Furthermore, BCA mitigated virus-induced weight loss compared to the virus control (Figure 9C). As shown in Figure 9D, oral administration of BCA (40 or 60 mg/kg) or molnupiravir (20 mg/kg) achieved a >99% reduction in viral loads in brain tissues at 7 dpi. Histopathological analysis via H&E staining revealed severe cerebral abnormalities in HCoV-OC43-infected mice, including nuclear shrinkage and degeneration (green arrows), cellular aggregation (red arrows), congestion (black arrows), and vacuolation (yellow arrows). These pathological changes were markedly attenuated by BCA or molnupiravir treatment (Figure 9G).

To confirm whether the AMPK/Nrf2/GSH pathway was upregulated in vivo, we analyzed brain tissue lysates by Western blot. BCA treatment at 40 and 60 mg/kg significantly increased the levels of p-AMPK, Nrf2, and its downstream mediator GCLC, while reducing viral replication (Figure 9E). Notably, BCA also elevated GSH content in the brain tissues (Figure 9F), further supporting that BCA exerts antiviral activity by upregulating the antioxidant pathway. Taken together, these in vivo results demonstrate that oral administration of BCA attenuates HCoV-OC43-induced encephalitis and protects infected mice by upregulating the AMPK/Nrf2/GSH signaling pathway and thus suppressing viral replication in the brain.

## 4. Discussion

The emergence of highly pathogenic coronaviruses such as SARS-CoV, MERS-CoV, and SARS-CoV-2 has underscored the persistent threat posed by this viral family to global health [1,23]. Beyond their impact on human health, other coronavirus infections also cause substantial economic losses in animal husbandry, as exemplified by PEDV, which induces severe enteric disease in piglets with mortality rates approaching 100% [24]. Although several anti-coronavirus drugs, including Molnupiravir and Paxlovid, were developed during the COVID-19 pandemic, they fall short of addressing the potential risks and challenges posed by diverse coronavirus subtypes infecting different hosts. Therefore, developing novel broad-spectrum anti-coronavirus agents is both imperative and urgent. Here, we report that Biochanin A (BCA) potently inhibits porcine coronavirus PEDV and human coronavirus HCoV-OC43 and HCoV-229E in vitro at non-cytotoxic concentrations. More importantly, oral BCA administration significantly improved survival and reduced brain viral loads of HCoV-OC43-infected mice. Mechanistically, we elucidate a novel mechanism whereby BCA activates the AMPK/Nrf2 pathway, boosting GSH synthesis to counteract viral oxidative stress and consequently inhibit its replication. To our knowledge, this study represents the first report of BCA’s anti-coronaviral activity and elucidates its underlying mechanism of action.

In biological systems, an imbalance between the production of ROS and the antioxidant defense system can result in cellular damage and physiological dysfunction, ultimately contributing to the development of various diseases [25]. Studies have shown that many viral infections disrupt redox homeostasis, leading to oxidative damage in cells and tissues and triggering inflammatory responses. For instance, influenza virus infection induces oxidative stress and contributes to pulmonary injury [26], while SARS-CoV-2 infection markedly upregulates oxidative stress-related genes and promotes ROS generation, thereby exacerbating pneumonia [27]. As reported by Kwon et al., HCoV-OC43 triggers oxidative stress via excessive ROS generation, ultimately leading to mitochondrial dysfunction [10]. The pathogenicity of PEDV has also been closely linked to elevated ROS levels. Specifically, PEDV activates the ERK signaling pathway to enhance ROS production, which plays a pivotal role in virus-mediated apoptosis and facilitates viral replication in host cells [11]. Consistent with these reports, we confirmed that PEDV infection induces ROS accumulation in Vero cells. Treatment with BCA effectively scavenged this virus-induced ROS, exhibiting an effect comparable to that of the known antioxidant xanthohumol. This antioxidant capacity is central to BCA’s antiviral function. BCA likely exerts its antioxidant effects through at least two complementary mechanisms: (1) direct ROS scavenging, which accounts for its rapid reduction of H_2_O_2_-induced ROS, and (2) indirect antioxidant activity via activation of the AMPK/Nrf2/GSH pathway, which enhances cellular antioxidant capacity through sustained GSH biosynthesis. In the context of PEDV infection, both mechanisms may contribute to the overall antiviral effect—direct scavenging provides immediate relief from virus-induced oxidative stress, while AMPK/Nrf2 activation establishes a prolonged antioxidant state that restricts viral replication. This interpretation is consistent with previous reports demonstrating that biochanin A possesses intrinsic ROS-scavenging capacity. The host transcription factor Nrf2 serves as the master regulator of the antioxidant response. Under oxidative stress, Nrf2 translocates to the nucleus and activates the expression of cytoprotective genes, including those involved in GSH synthesis [28]. Our data demonstrate that BCA treatment significantly upregulated Nrf2 expression and increased its nuclear translocation. The critical role of Nrf2 in mediating BCA’s antiviral activity was further supported by experimental evidence: overexpression of Nrf2 effectively inhibited PEDV replication, whereas suppression of Nrf2—either via siRNA knockdown or through pharmacological inhibition with hinokitiol—completely abolished the protective effect of BCA.

We further investigated the upstream signaling events leading to Nrf2 activation. The activity of Nrf2 is known to be regulated by several key upstream pathways. First, AMP-activated protein kinase (AMPK) can directly phosphorylate Nrf2, thereby promoting its nuclear translocation and transcriptional activity [13]. Second, GSK-3β exerts negative regulation on Nrf2; its phosphorylation and inactivation lead to the stabilization and subsequent activation of Nrf2 [29,30]. Furthermore, the p62 protein can compete with Nrf2 for binding to its negative regulator, Keap1, thereby facilitating Nrf2 stabilization and activation [31,32]. Given the roles of these pathways, we evaluated the effect of BCA on AMPK, GSK3β, and p62 in our study. The results showed that BCA treatment enhances AMPK phosphorylation, and knockdown of AMPK expression abolishes BCA-mediated Nrf2 upregulation and antiviral activity. The specificity of this pathway was supported by the observation that BCA had no significant effect on other known Nrf2 regulators, such as GSK3β and p62. The functional importance of the AMPK/Nrf2 pathway was further confirmed by its consistent activation in the brains of HCoV-OC43-infected mice treated with BCA, which correlated with reduced viral replication and improved survival. This concordance between in *vitro* and in *vivo* findings strongly suggests that AMPK/Nrf2 activation constitutes the core mechanism underlying the broad-spectrum anti-coronavirus activity of BCA. To explore whether BCA directly interacts with AMPK, we performed a cellular thermal shift assay (CETSA) and molecular docking analysis. Our CETSA results showed that BCA treatment did not alter the thermal stability of AMPK (Supplementary Figure S1A), while molecular docking revealed a low binding affinity (−6.5 kcal/mol) with no stable hydrogen bond interactions between BCA and the AMPK active site (Supplementary Figure S1B). These findings suggest that BCA does not directly bind to AMPK to activate its phosphorylation. AMPK activation can be triggered by several distinct mechanisms, including upstream kinases such as LKB1 [33] and CaMKKβ (Ca^2+^/calmodulin-dependent protein kinase β) [34], as well as changes in cellular energy status [20] and mitochondrial function [35]. The precise mechanism by which BCA enhances AMPK phosphorylation—whether it involves direct interaction with AMPK, modulation of these upstream kinases, or alterations in mitochondrial function and cellular energy homeostasis—remains to be determined and is critical for elucidating the initiating event of its antiviral signaling cascade.

Furthermore, network pharmacology analysis predicted that pathways such as PI3K-Akt may be involved in the anti-coronavirus activity of BCA. Although the present study focused on the AMPK/Nrf2/GSH pathway and our experimental data suggest that BCA does not affect GSK3β, a downstream component of the PI3K-Akt pathway, the potential contribution of other pathways requires further experimental validation. This constitutes a limitation of the current study and represents an important direction for our future research. Nrf2 regulates an extensive network of cytoprotective genes, including those encoding heme oxygenase-1 (HO-1), glutathione synthetase (GSS), NAD(P)H quinone dehydrogenase 1 (NQO1), and superoxide dismutase 1 (SOD1), which collectively orchestrate the cellular antioxidant defense. A key finding of this study was the identification of the specific metabolic effector downstream of Nrf2 responsible for mediating the antiviral effects of BCA. Among its numerous targets, BCA specifically and dose-dependently upregulated the expression of GCLC—the rate-limiting enzyme in glutathione (GSH) synthesis. Consequently, BCA treatment increased intracellular GSH levels. The critical role of GSH in suppressing PEDV replication was supported by multiple lines of evidence: exogenous GSH or its precursor NAC inhibited viral replication, whereas the GSH synthesis inhibitor buthionine sulfoximine (Bso) promoted it. Most importantly, Bso reversed the antiviral effect of BCA, directly linking BCA’s effect to its ability to promote GSH biosynthesis. Together, these results establish the AMPK/Nrf2/GSH pathway as the definitive signaling cascade through which BCA exerts its host-directed antiviral activity.

Elevated GSH levels exert antiviral effects through multiple interconnected pathways. As the primary intracellular antioxidant, GSH helps maintain redox homeostasis and counteracts virus-induced oxidative stress, which is exploited by many coronaviruses, including PEDV, to promote replication. Beyond its redox-regulating role, GSH also modulates key antiviral processes: it suppresses nuclear factor-kappa B (NF-κB) activation, potentially attenuating excessive inflammation triggered by infection [36]; it inhibits virus-induced apoptosis, limiting virion release from dying cells [37]; and evidence suggests it may directly disrupt viral replication, possibly by altering intracellular pH or other replication-essential conditions [38].

PEDV primarily infects small intestinal epithelial cells, leading to villous atrophy and disruption of the mucosal barrier. These pathological changes lead to diarrhea, dehydration, and high mortality in piglets. Notably, the intestinal damage and inflammatory responses induced by PEDV infection share significant pathophysiological features with dextran sulfate sodium (DSS)-induced colitis models, including impaired barrier function, upregulation of pro-inflammatory cytokines, and immune cell infiltration [39]. This overlap suggests that compounds effective against DSS-induced colitis may also alleviate PEDV-induced intestinal injury. Supporting this notion, Chen et al. reported that a PEDV protease inhibitor (compound f2), which exhibits in vitro antiviral activity (EC_50_ = 1.17–2.02 μM), ameliorated DSS-induced colitis in mice [39]. Importantly, BCA has demonstrated comparable efficacy in the same DSS-induced colitis model, where oral administration (40 mg/kg) significantly alleviated disease severity [40]. Given that BCA both directly inhibits PEDV replication via AMPK/Nrf2 activation and preserves intestinal barrier integrity, as evidenced in DSS colitis, it is reasonable to anticipate therapeutic benefits of BCA in PEDV-infected piglets. Future studies should prioritize in vivo evaluation of BCA in PEDV-challenged piglets, with emphasis on viral shedding reduction, restoration of villous structure, and activation of the AMPK/Nrf2 in intestinal tissues.

The development of coronavirus therapeutics highlights distinct advantages of host-directed over virus-targeted approaches. Direct-acting antivirals, though specific, are vulnerable to rapid resistance caused by viral mutations—a limitation evident during the COVID-19 pandemic, where the emergence of highly mutated SARS-CoV-2 variants raised concerns about the diminished potency of several early virus-targeted agents [41,42,43]. In contrast, host-directed agents like BCA target genetically stable host pathways essential for viral replication, thereby offering a higher barrier to resistance. Moreover, since such pathways are often exploited by multiple coronaviruses, this strategy possesses inherent broad-spectrum potential. The ability of BCA to inhibit both *alpha*- and *beta*-coronaviruses in our study supports this notion, suggesting its utility against future emerging coronaviruses reliant on similar host mechanisms. Thus, BCA represents a promising host-directed antiviral with the versatility to address both current and future outbreaks.

## 5. Conclusions

**Figure 10:**
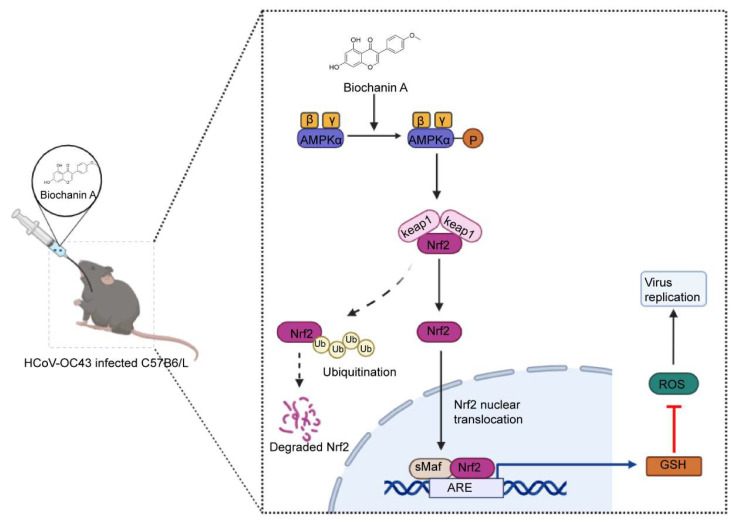
Schematic representation of the antiviral mechanism of BCA. BCA upregulates the AMPK/Nrf2 signaling pathway, promoting Nrf2 nuclear translocation and increasing GSH expression. This activation of antioxidant defense reduces virus-induced ROS accumulation, thereby exerting an antiviral effect.

Our findings identify BCA as a broad-spectrum anti-coronavirus agent that operates through a well-defined host-directed mechanism. By activating the AMPK/Nrf2/GSH signaling pathway, BCA enhances cellular antioxidant defenses, thereby establishing an intracellular environment that is unfavorable to viral replication. The mechanism is illustrated in Figure 10. Together with its natural origin, favorable safety profile, and efficacy in a lethal infection model, BCA represents a promising lead compound for developing novel therapeutics against emerging coronaviruses.

## Figures and Tables

**Table 1:** Specific primer sequences for qRT-PCR analysis.

Name	Sequences 5′ to 3′
PEDV-N-F	*5′-CGCAAAGACTGAACCCACTAATTT-3′*
PEDV-N-R	*5′-TTGCCTCTGTTGTTACTTGGAGAT-3′*
HCoV-OC43-N-F	*5′-AGCAACCAGGCTGATGTCAATACC-3′*
HCoV-OC43-N-R	*5′-AGCAGACCTTCCTGAGCCTTCAAT-3′*
HCoV-229E-N-F	*5’-TAGGTTTTGACAAGCCTCAGGAAAAAGA-3’*
HCoV-229E-N-R	*5’-GTGACTATCAAACAGCATAGCAGCTGT-3’*
Huh7-GAPDH-F	*5’-GCACCGTCAAGGCTGAGAAC-3’*
Huh7-GAPDH-R	*5’-TGGTGAAGACGCCAGTGGA-3’*
Vero-GAPDH-F	*5’-GGACTTCGAGCAGGAGATGG-3’*
Vero-GAPDH-R	*5’-AGGAAGGAGGGCTGGAAGAG-3’*

## Data Availability

The data generated during this study are available from the corresponding authors upon reasonable request.

## Supplementary Materials

The following supporting information can be downloaded at: https://www.mdpi.com/article/10.3390/microorganisms14040851/s1, Figure S1: Evaluation of direct binding between BCA and AMPK; Table S1: Cytotoxicity of six natural flavonoids on Vero cells after 48 h of treatment with CC10 and CC50 values.
