# Supplementary material for: Biochanin A Exerts Broad-Spectrum Antiviral Activity Against Coronaviruses via Activating the AMPK/Nrf2/GSH Pathway
Source: Microorganisms. 2026 Apr 9;14(4):851. doi: 10.3390/microorganisms14040851 (PMC13118608; doi:10.3390/microorganisms14040851)
Supplement: Supplementary file 1 [file microorganisms-14-00851-s001.zip › microorganisms-4193124-supplementary.pdf]

Supplementary Table S1.

Table S1 Cytotoxicity of six natural flavonoids on Vero cells after 48 h treatment with CC10 and CC50 values

Compound	CC_{10} (μM)	CC_{50} (μM)
Genistein	111	261
Daidzein	88.2	207
Glycitein	43.6	128
Sophoricoside	80.4	248
Isoflavone	36.8	95.8
Biochanin A (BCA)	85.4	271

Supplementary Figure S1.

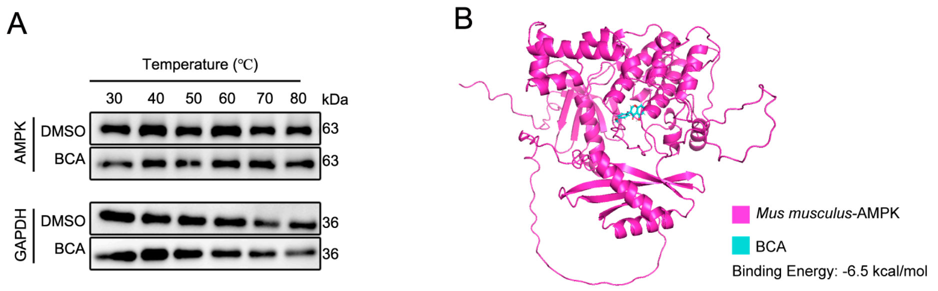

Figure S1. Evaluation of direct binding between BCA and AMPK. **(A)** Vero cells were treated with BCA (15 μM) or DMSO control for 1 h, followed by heating at the indicated temperatures. Cell lysates were analyzed by Western blotting using an anti-AMPK antibody. **(B)** Molecular docking analysis. The three-dimensional structure of AMPK was used to simulate the binding mode of BCA. Molecular docking was performed to predict potential interactions between BCA and the AMPK active site.
